# Effects of bodybuilding supplements on the kidney: A population-based incidence study of biopsy pathology and clinical characteristics among middle eastern men

**DOI:** 10.1186/s12882-020-01834-5

**Published:** 2020-05-06

**Authors:** Alaa Abbas Ali, Safaa E. Almukhtar, Dana A. Sharif, Zana Sidiq M. Saleem, Dana N. Muhealdeen, Michael D. Hughson

**Affiliations:** 1 Department of Pathology, University of Sulaimani College of Medicine and Shoresh Teaching Hospital, Quirga Road, Sulaimani, Iraq; 2 Department of Nephrology, University of Hawler College of Medicine, Erbil, Iraq; 3 Department of Nephrology, University of Sulaimani College of Medicine, Sulaimani, Iraq; 4 Department of Nephrology, University of Dohuk College of Medicine, Dohuk, Iraq

**Keywords:** Acute kidney injury, Bodybuilding, Nephrocalcinosis, Anabolic steroids, Supplements, Focal segmental glomerulosclerosis

## Abstract

**Background:**

The incidence of kidney diseases among bodybuilders is unknown.

**Methods:**

Between January 2011 and December 2019, the Iraqi Kurdistan 15 to 39 year old male population averaged 1,100,000 with approximately 56,000 total participants and 25,000 regular participants (those training more than 1 year). Annual age specific incidence rates (ASIR) with (95% confidence intervals) per 100,000 bodybuilders were compared with the general age-matched male population.

**Results:**

Fifteen male participants had kidney biopsies. Among regular participants, diagnoses were: focal segmental glomerulosclerosis (FSGS), 2; membranous glomerulonephritis (MGN), 2; post-infectious glomeruonephritis (PIGN), 1; tubulointerstitial nephritis (TIN), 1; and nephrocalcinosis, 2. Acute tubular necrosis (ATN) was diagnosed in 5 regular participants and 2 participants training less than 1 year. Among regular participants, anabolic steroid use was self-reported in 26% and veterinary grade vitamin D injections in 2.6%. ASIR for FSGS, MGN, PIGN, and TIN among regular participants was not statistically different than the general population. ASIR of FSGS adjusted for anabolic steroid use was 3.4 (− 1.3 to 8.1), a rate overlapping with FSGS in the general population at 2.0 (1.2 to 2.8). ATN presented as exertional muscle injury with myoglobinuria among new participants. Nevertheless, ASIR for ATN among total participants at 1.4 (0.4 to 2.4) was not significantly different than for the general population at 0.3 (0.1 to 0.5). Nephrocalcinosis was only diagnosed among bodybuilders at a 9-year cumulative rate of one per 314 vitamin D injectors.

**Conclusions:**

Kidney disease rates among bodybuilders were not significantly different than for the general population, except for nephrocalcinosis that was caused by injections of veterinary grade vitamin D compounds.

## Background

Weight training has become popular throughout much of the world, and it is estimated that 15–20% of United States (US) and European populations are members of gymnasiums [1]. Most gymnasiums sell supplements that typically consists of protein powders, creatine, and oral vitamins and minerals. Credible gymnasiums will not market anabolic steroids, but they are readily available in the outside community. The injection of subcutaneous and intramuscular high dose veterinary vitamin D and K compounds is practiced in South America and the Middle East with an apparently high frequency of end-stage renal disease (ESRD) [2–4].

A majority of competitive bodybuilders and weight athletes are likely to use anabolic steroids even though they are banned from most organized sports [5]. In 2013, the current superheavyweight Worlds Weight Lifting Champion and more than 100 other weight lifters from virtually every region of the world served bans after being tested positive for anabolic steroids [6]. Focal segmental glomerulosclerosis (FSGS) is frequently attributed to both anaboloic steroids and excess protein intake [7–9]. Nevertheless, the scale of the risk of FSGS, or any kidney disease, with bodybuilding supplements compared to the general population is currently undetermined.

The Kudistan region in Northern Iraq has established nephrology practices and a centralized renal biopsy service [10]. We have developed a particular interest in supplement induced kidney injury, because several otherwise healthy young men have been identified with acute and chronic renal disease whose common background was a participation in gymnasium affiliated bodybuilding and the use of supplements [11]. This current population-based renal biopsy study estimates the incidence of specific diagnoses of renal disease among bodybuilders and compares the incidence to age matched, biopsy-defined kidney disease in the general population.

## Methods

The study was observational for two defined periods of time. One for bodybuilders and one for the general population. The inclusion criteria were a renal biopsy for both groups, and there were no exclusion criteria. While this was a review of existing data and not a true cross-sectional study, the STROBE reporting checklist for cross-sectional studies was followed for items that seemed appropriate. This included the methods of estimating population sizes and precision estimates of the quantitative variable of age specific incidence rate (ASIR).

Bodybuilders and patients from the general population were residents of Sulaimania, Erbil, or Dohuk and were biopsied because of an elevated serum creatinine and/or proteinuria. All patients completed a supplement use questionaire and gave written permission for use of their information and biopsy results. All biopsies were studied by light microscopy in 18 serial sections using hematoxylin and eosin, periodic acid-Shiff, Masson trichrome, and Jones methenamine silver stains, and by immunofluorescence microscopy with fluorescein conjugated anti-human IgG, IgM, IgA, C3, C1q, and albumin. Electron microscopy was not performed on any of the bodybuilding cases.

### Estimates of the bodybuilding population and the frequency of supplement use

The number of regional bodybuilders was tabulated from 2019 client registration lists by managers of gymnasiums in Erbil, Sulaimania, and Dohuk and extrapolated to the number of gymnasiums registered in 2015 by the Kurdistan Regional Government Licensing Committee. A bodybuilder was defined as any persons participating in weight training at regional gymnasiums. The gymnasium registrations were divided into two categories. Category A: all registrants. Category B: regular registrants who participated in weight training for more than 1 year. The average annual retention rate for newcomers at major gymnasiums was 44%. For the year 2015, all registrants were estimated at 56,000 and regular registrants at 25,000. The ages of 94% of the registrants were between 18 and 39 years old. Gymnasium use is gender restricted and offers machine weights, free weights, cycling machines, and treadmills.

To determine the frequency of training and rate of supplement use among gymnasium participants, questionnaires developed by DAS and DNM were filed out by 150 gymnasium users at three major gymnasiums in Sulaimania in the presence of investigator DNM and gymnasium supervisors. Informed consent to participate was indicated in writing by a coded initial on the questionaire form. Completion of the questionnaire was followed by an exit interview, and the compiled data was reviewed by the gymnasium supervisors. Participants at all level of training took part in the questionnaire and interview process, i.e. the process was not directed toward more or less experienced trainees.

### ASIR estimates in the general population

The annual ASIR of kidney biopsy diagnoses per 100,000 males among the general Kurdistan population was calculated for the 2 year period 2012–2013 as previously reported [10]. An estimate of the Kurdistan population at 4,900,000 persons was derived from 2011 to 2012 United Nations Iraq population data and the 2012 Iraqi Cancer Registry [12]. The estimated number of males between 15 to 39 years of age was 1,100,000.

### ASIR estimates of kidney disease among bodybuilders

Kidney biopsies were obtained on 15 bodybuilders over a 9.0 year period from January 1, 2011 to December 31, 2019. The annual ASIR of kidney biopsy disease diagnoses per 100,000 bodybuilders was calculated from 25,000 regular participants for FSGS, membranous glomerulonephritis (MGN), post-infectious glomerulonephritis (PIGN), tubulointerstitial nephritis (TIN), and nephrocalcinosis because all of these biopsies were obtained from trainees with more than 1 year experience. For acute tubular necrosis (ATN), ASIR was calculated from all participants at 56,000, because not all ATN patients were regular participants. ASIR for bodybuilders was compared with the rates of disease in the general population in the years 2012–2013. Because of the small number of bodybuilding cases, the aggregate age range of 15 to 39 years was used to calculate ASIR for both bodybuilders and the general population.

### Comparisons of ASIR between bodybuilders and the general population

The precision estimates of ASIR were determined by 95% confidence intervals (95% CI) for specific biopsy diagnoses. The ASIR of each diagnosis was calculated as: 95% CI (ASIR) = 1.96x (√Ri^2^/Ni); where, Ri = age-specific incidence in the 15–39 year old age group and Ni = number of biopsies in the 15–39 year age group [13]. Differences in ASIR between bodybuilders and the general population were considered significant if the 95% CI did not overlap.

## Results

### Bodybuilding patients: clinical findings and renal biopsy results

The clinical characteristics, selected laboratory data, biopsy findings, and supplement use of the 15 bodybuilders are summarized in Table 1. The diagnoses consisted of seven cases of ATN, one case of TIN, two cases of FSGS, one case of PIGN, two cases of MGN, and two cases of nephrocalcinosis.

**Table 1:** Clinical characteristics, laboratory data, and pathology of regional bodybuilders (all male) undergoing renal biopsies

Patient no.	Age	Duration of training	Pathology diagnosis	IF/TA	AS	protein	creatine	Vit D use	S (CK)	S (Ca)	S (Cr)	S (Cr) 1 year
1	26	7 years	ATN, microcalcifications	30%	+	+	+	Oral	nd	10.1	2.8	1.3
2	21	3 years	ATN, microcalcifications	15%	+	+	+	Oral	nd	10.0	3.8	1.0
3	21	4 years	ATN, microcalcifications	0	+	+	+	Oral	nd	9.4	3.1	1.1
4	20	3 years	ATN, microcalcifications	40%	+	+	+	Oral	nd	10.0	2.6	1.2
5	23	4 years	ATN, microcalcifications	20%	+	+	+	Oral	nd	9.8	3.2	1.2
6	22	6 month	ATN, myoglobin casts	30%	–	+	–	–	6893	nd	8.6	1.0
7	19	1 month	ATN, myoglobin casts	0	–	–	–	–	1254	nd	3.8	1.0
8	30	5 years	TIN	10%	–	+	+	Oral	nd	8.9	3.4	1.1
9	30	10 years	FSGS, NOS	50%	+	+	+	Oral	nd	nd	2.5	ESRD
10	40	20+ years	FSGS, perihilar	0	+	+	+	Oral	nd	nd	1.3	1.3
11	32	8 years	PIGN	0	+	+	+	Oral	nd	nd	1.9	1.2
12	49	20+ years	MGN	5%	+	+	+	Oral	nd	nd	1.4	1.1
13	26	5 years	MGN	25%	+	+	+	Oral	nd	nd	1.6	1.3
14	27	8 years	Nephrocalcinosis	70%	+	+	+	INJ	nd	13.1	1.9	ESRD
15	24	2 years	Nephrocalcinosis	40%	+	+	+	INJ	nd	12.8	2.0	3.4

*Abbreviations*: *nd* not done, *IF/TA* interstitial fibrosis and tubular atrophy, *AS* anabolic steroids, *S (Ca)* serum calcium, mg/dL, *S (Cr)* serum creatinine, mg/dL, *S (CK)* serum creatine kinase, U/L, *ATN* acute tubular necrosis, *FSGS* focal segmental glomerulosclerosis, *PIGN* post-infectious glomerulonephritis, *MGN* membranous glomerulonephritis, *TIN* tubulointerstitial nephritis, *INJ* injected veterinary grade Vitamin D compounds, *ESRD* end-stage renal disease. Laboratory data is for the time of diagnosis except for serum creatinine at one year follow-up [S (Cr) 1 year]

### Biopsies of acute tubular necrosis (ATN)

Two of the seven patients (patients 6 and 7) with ATN presented with muscle pain and elevated serum creatine kinase levels at one and 6 months after starting bodybuilding. The biopsies revealed degenerative and regenerative epithelium lining dilated tubules that contained immunohistochemically demonstrated myoglobin casts.

**Fig. 1:**
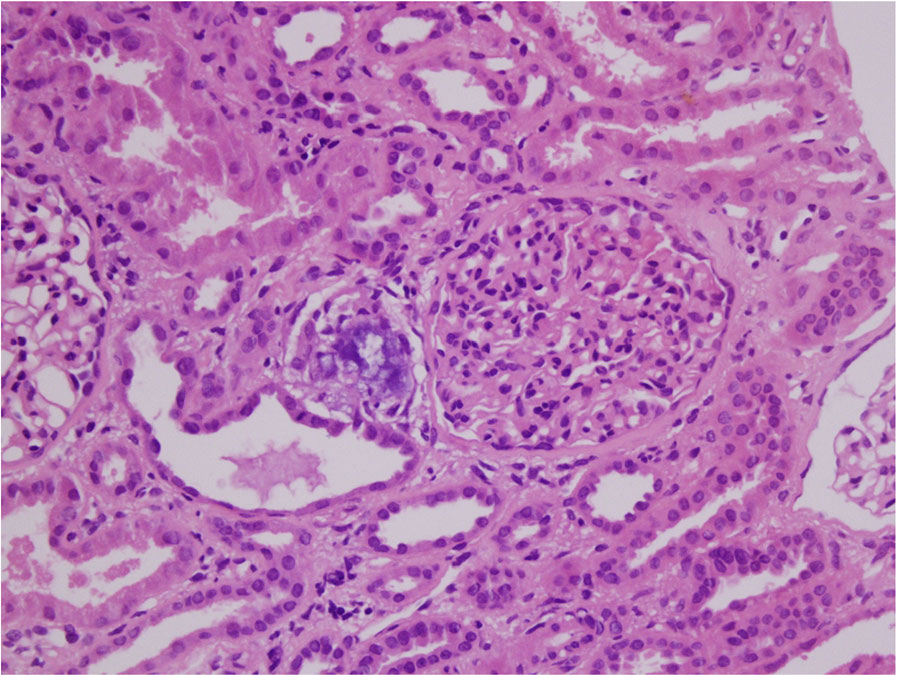
Patient 1. A 26 year old who had been training for 36 months. He presented with renal insufficiency and a creatinine of 2.6 mg/dl. The biopsy shows dilated tubules lined by flattened mitotically active epithelium. An intraluminal calcification surrounded by regenerative cells is present. Hematoxylin and eosin stain × 400

Five of the seven ATN patients had been bodybuilding from 2 to 7 years and presented with afebrile malaise but not muscle pain, and creatine kinase levels were not obtained. The biopsies of these five patients showed intratubular microcalcifications associated with foci of degenerating and regenerating tubular epithelium (Fig. 1) but with no myoglobin casts.

Five of the seven ATN biopsies revealed evidence of chronic injury with 15–40% interstitial fibrosis and tubular atrophy. In all ATN patients, supplement use was discontinued, and with no additional treatment except IV fluids, serum creatinine levels returned to normal. The clinical ATN events occurred during the summer in four patients and during the winter in three patients, and all training took place in air conditioned gymnasiums.

### Biopsy of tubulointerstitial nephritis (TIN)

The biopsy of patient 8 revealed a TIN with lymphocytic and plasma cell infiltrates but without eosinophils. There was no pyelographic evidence of reflux, and the biopsy showed minimal chronicity. The renal failure resolved when supplements were discontinued.

### Biopsies of focal segmental glomerulosclerosis (FSGS)

The biopsy of patient 9, a 30 year old, with FSGS showed a not otherwise specified pattern with four of five glomeruli showing segmental or global glomerulosclerosis and with more than 50% interstitial fibrosis and tubular atrophy. Immunofluorescence (IF) microscopy revealed focal segmental glomerular IgM and C3 staining. Transplantation was required 36 months following the diagnosis.

The biopsy of the patient 10, a 40 year old, demonstrated perihilar FSGS with hyalinosis in one of 8 glomeruli and focal glomerular and vascular C3 staining by IF. No interstitial fibrosis or tubular atrophy was seen. This patient was a competitive bodybuilder with a lean body mass index (BMI) of 33.6. He had a 20 year history of anabolic steroid and protein supplement use. At 1 year after the biopsy, a 24 h urine collection demonstrated 1.23 g of protein with a glomerular filtation rate of 82.6 ml/min/1.73m^2^.

### Biopsies of post-infectious and membranous glomerulonephritis (PIGN and MGN)

The biopsy of patient 11 demonstrated diffuse and global endocapillary glomerular hypercellularity with neutrophils and occasional eosinophils. IF showed a starry sky pattern of staining for IgG and C3 that was consistent with PIGN. The patient had no signs of any injection site infection, and cardiac ultrasonography showed no valvular disease. The serum creatinine returned to a normal range, and at 6 months after the biopsy, urine was negative for protein. The biopsies of patients 12 and 13 were consistent with a primary MGN showing diffuse glomerular basement membrane thickening with no appreciable mesangial hypercellularity. IF demonstrated granular glomerular capillary loop staining for IgG and C3. Serological testing for ANA and dsDNA were negative; antibodies for anti-phospholipase A2 receptor testing were not available.

### Biopsies of nephrocalcinosis

**Fig. 2:**
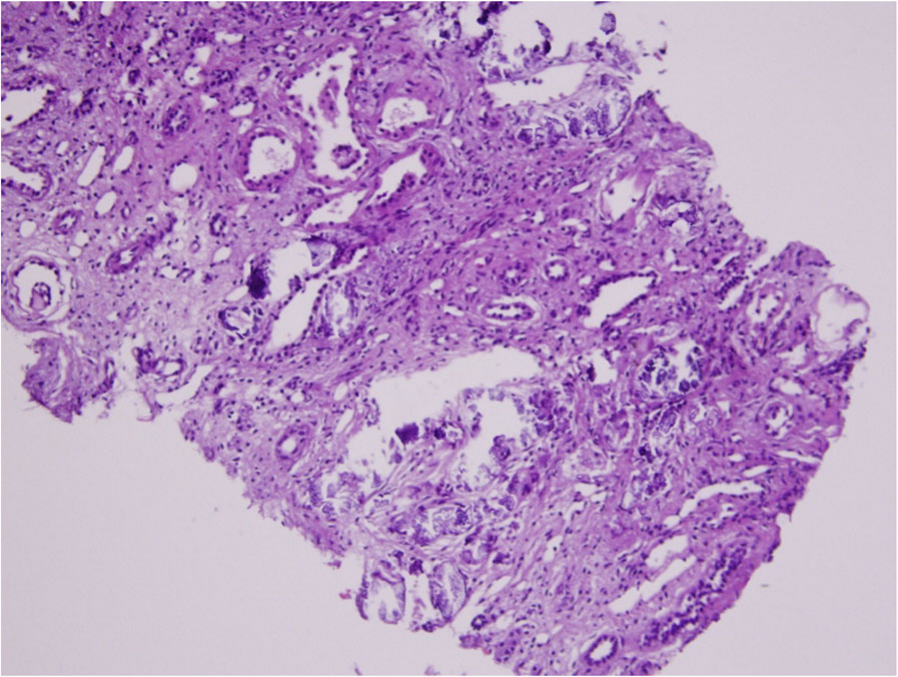
Patient 14. A 27 year old competitive body builders who injected veterinary grade vitamin compounds. The biopsy shows massive tubular and interstitial calcium deposits with advanced interstitial fibrosis and tubular atrophy. He required transplantation shortly after the biopsy. Hematoxylin and eosin stain × 100

Patients 14 and 15 injected veterinary grade 100 ml solutions containing 50,000,000 IU of vitamin K, 7,000,000 IU of vitamin D, and 5000 IU of vitamin E in a sesame oil base. The kidney biopsies demonstrated extensive intratubular and interstitial calcium deposits and advanced interstitial fibrosis and tubular atrophy (Fig. 2). Patients 14 progressed to ESRD and kidney transplantation shortly after biopsy. At the last clinic visit, patient 15 had a serum creatinine of 3.4 mg/dl with a Chronic Kidney Disease Epidemiology Collaboration (CKD-EPI) eGFR of 23.9 ml/min/1.73m^2^ .

**Fig. 3:**
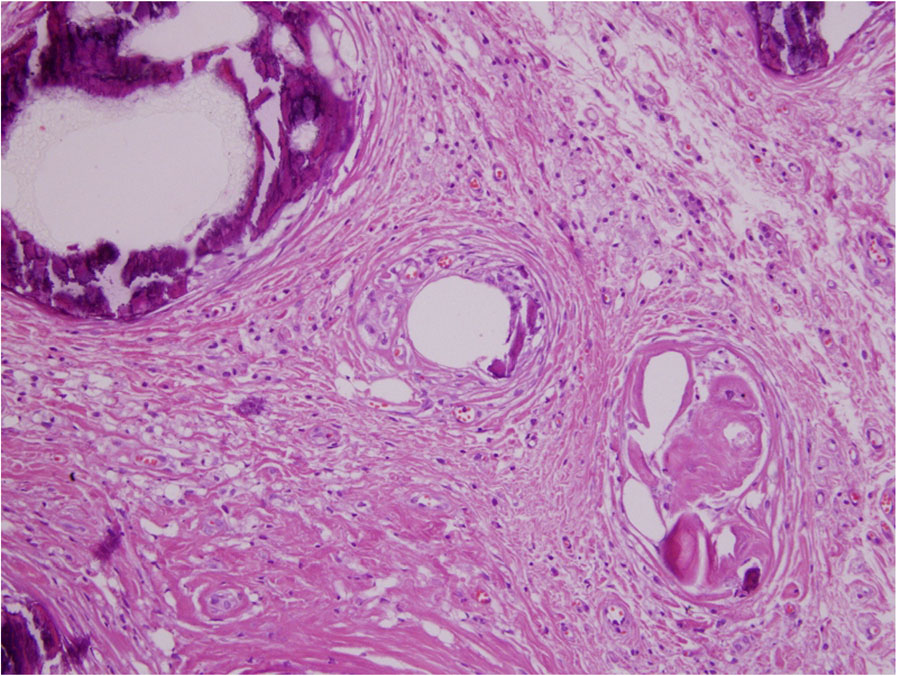
Patient 15. Soft tissue removed from veterinary grade vitamin injection sites in the shoulder of a 24 year old bodybuilder. The soft tissue was removed 20 months after a kidney biopsy showed nephrocalcinosis. At this 20 month interval, he was hypercalcemic and had hypervitaminosis D. The serum creatinine had increased to 3.4 mg/dl from 1.9 mg/dl at the time of his renal biopsy. The tissue demonstrates calcifications and lipogranulomatous inflammation surrounding oil droplets. Hematoxylin and eosin stain × 100

Soft tissue was removed from the shoulder, chest, and arm injection sites of the two patients with nephrocalcinosis. This tissue showed lipogranulomatous inflammation with large calcium deposits (Fig. 3). When the soft tissue was removed 20 months after the kidney biopsy of patient 15, serum calcium was 12.8 mg/dl (normal range 8.5 to 10.5 mg/dl) and serum vitamin D was 158 ng/ml (normal range 30–80 ng/ml).

### Incidence estimates of kidney disease diagnoses among body builders

Questionaires completed by 150 gymnasium participants recorded the following: 82 (55%) were were gymnasium members less than 1 year, and 68 (45%) had been members for more than a year. All participated in a combination of free and machine weight training. For the first year members, anabolic steroid use was 7% and gymnasium participation averaged 3.5 ± 1 days a week. None of the first year members reported human growth hormone (hGH) or veterinary vitamin D injections. For regular members, gymnasium usage was 5.4 ± 1 days a week and the reported supplement usage was: Anabolic steroids, 26%; protein powders, 86%; creatine, 79%; injected high dose veterinary vitamin D, 2.6%; and hGH, 5%.

For the 26% of regular members using anabolic steroids, combinations of stanozolol and nandrolone were reported by 11%, testosterone proprionate/cypionate and stanozolol/nandrolone by 10%, and a single agent either testosterone or nandrolone by 6%. The most common anabolic steroid combination was 250 mg injections of nandrolone twice a week and a 250 mg injection of stanozolol once a week. Hyperphysiological daily dosing of multiples of 250 mg for any agent was not recorded. Gymnasium supervisors stated that the use non-steroidal anti-inflammatory drugs and diuretics is virtually non-existent among regional bodybuilders.

**Fig. 4:**
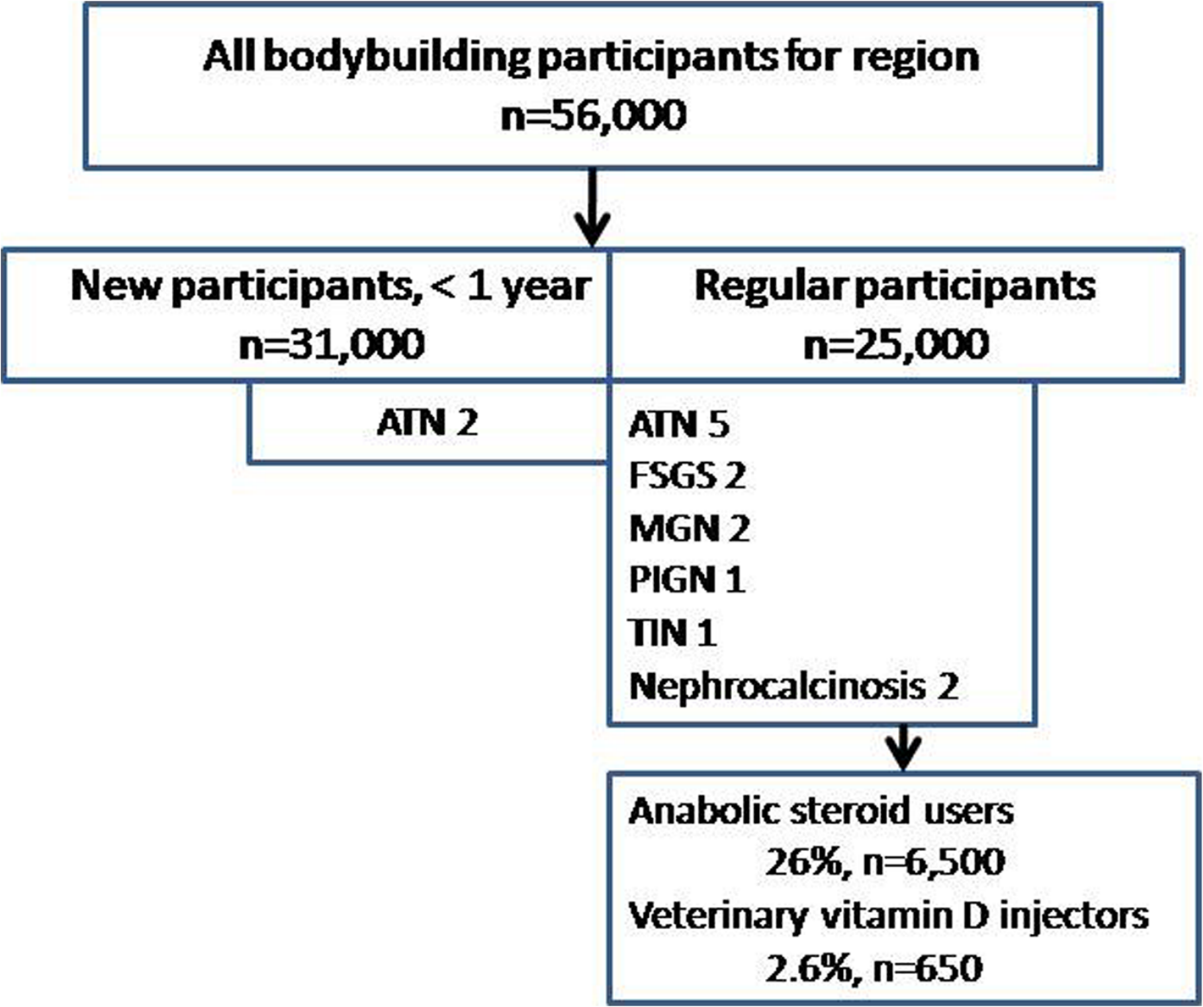
Flow chart of the bodybuilding population, indicating estimated populations, biopsy diagnoses, and risk-associated supplement use

Figure 4 is a flow chart that provides the population estimates of all participants and regular participants. Out of 56,000 total gymnasium participants, there were an estimated 25,000 regular participants, 6500 anabolic steroid users, and 650 vitamin D injectors.

Table 2 shows the ASIR of renal biopsy diagnoses among bodybuilders in which the annual biopsy rate was 5.8 per 100,000 regular participants. For regular participants, ASIR for FSGS, MGN, and nephrocalcinosis was 0.9 (− 0.4 to 2.2), and for PIGN and TIN, ASIR was 0.5 (− 0.4 to 1.4). The ASIR for FSGS, MGN, PIGN, and TIN were similar to those for the general population (Table 3). When adjusted for the 26% of anabolic steroid use, the ASIR of FSGS among body builders was 3.4 (− 1.3 to 8.1). Nevertheless, an absence of statistical significance was reflected in the wide 95% CI that overlapped the incidence of FSGS in the general population at 2.0 (1.2 to 2.8). The annual biopsy rate in the general population for the diseases found among body builders was 3.4 per 100,000 15 to 39 year old males.

**Table 2:** Age specific incidence rate (ASIR) of kidney disease diagnosed among bodybuilders in Kurdistan of Iraq in the 9 year period from January 1, 2011 through December 31, 2019

Diagnosis	Biopsies 9.0 yrs	Annual average	Pop at risk	ASIR	95% CI	ASIR by suppl	95% CI by suppl
FSGS	2	.22	25,000	0.9	−0.3 to 2.1	3.4	−1.3 to 8.1
MGN	2	.22	25,000	0.9	−0.3 to 2.1		
PIGN	1	.11	25,000	0.4	−0.4 to 1.3		
TIN	1	.11	25,000	0.4	−0.4 to 1.3		
ATN	7	.78	56,000	1.4	0.4 to 2.4		
nephrocalcinosis	2	.22	25,000	0.9	−0.3 to 2.1	33.8	−13.0 to 80.6

ASIR was calculated per 100,000 body builders for all diagnoses and adjusted by specific supplement use for FSGS (anabolic steroids, 26%) and nephrocalcinosis (injected vitamin D, 2.6%). The population at risk for bodybuilders was considered to be regular participants at 25,000 except for ATN in which the population at risk was considered to be all participants at 56,000

**Table 3:** Age specific incidence rate (ASIR) of kidney disease diagnosed among the male general population of Kurdistan of Iraq aged 15 to 39 years old in the two years period 2012–2013

Diagnosis	Biopsies 2 yrs	Annual average	Pop at risk	ASIR	95% CI
FSGS	45	22.5	1,100,000	2.0	1.2 to 2.8
MGN	17	8.5	1,100,000	0.8	0.3 to 1.3
PIGN	2	1	1,100,000	0.1	0.0 to 0.2
TIN	7	3.5	1,100,000	0.3	0.0 to 0.6
ATN	6	3	1,100,000	0.3	0.1 to 0.5
Nephrocalcinosis	0	0	1,100,000	0	

ASIR was calculated per 100,000 males

Because two patients with ATN were newcomers to bodybuilding, the ASIR for ATN was calculated from the number of all gymnasium registrants at 1.4 (0.4 to 2.4). The overlapping with ATN in the 15 to 39 year old general male population at 0.3 (0.1 to 0.5) indicates that the frequencies of ATN were not statistically different for the two groups.

The use adjusted ASIR for nephrocalcinosis was 33.8 (− 13.0 to 80.6) per 100,000 vitamin D injectors. There was no group for comparison in the general population.

## Discussion

Among Iraqi Kurdistan bodybuilders, renal disease rates, except for nephrocalcinosis, were similar to those found in the age-matched, general male population. Nephrocalcinosis was a uniquely bodybuilding disease and was found only with injections of veterinary grade vitamin D compounds. It did not occur in everyone using veterinary compounds but had an estimated 9-year cumulative occurrence of one per 314 vitamin D injectors. Injecting patients can present with reversible acute kidney injury, but once nephrocalcinosis is established, the outcome appears ominous with a risk of ESRD that from the limited Kurdistan experience may approach 50% within 2 years after diagnosis [3, 4].

ATN was the most common renal disease encountered among our bodybuilders. We commonly see elevated creatinine levels in laborers and soldiers during the summer months, but these patients are treated on the basis of clinical and laboratory findings and are not biopsied. Patients with evidence of AKI are usually biopsied if there is no apparent underlying cause, and these biopsied patients comprise our estimates of the incidence of ATN in the general 15–39 year old male population.

Data on the rates of AKI among young males is not readily available, but the 1996 study by Liano et al. from Madrid [14] reports an annual clinical incidence for ATN of 8.8 patients per 100,000 persons in which the average patient age was 63 ± 17 years. The proportion of AKI patients under 44 years old with no co-morbid disease has been estimated at 10.5% [15]. That frequency would calculate to an annual incidence of 0.9 (0.6 to 1.2) young Madrid patients per 100,000 residents [14]. While this is higher that than the biopsy incidence of ATN at 0.3 (0.1 to 0.5) per 100,000 among the general Kurdish male population, the difference is not particularly great and shows, even with the disparity between clinical and biopsy diagnoses, that AKI is uncommon among otherwise healthy young males.

Two of the ATN patients that we report were new to bodybuildng and presented with muscle pain and evidence of rhabdomyolysis. This is referred to as exertional muscle injury and is attributed to microscopic muscle damage [16, 17]. It is sometimes accompanied by myoglobinuria that is of concern because of the association of myoglobinuria with kidney injury [17].

The biopsies of ATN among the more experienced bodybuilders contained microcalcifications, and all patients consumed commercial vitamin and mineral capsules as well as protein and creatine powders. Nevertheless, all of this consumption was well within amounts that, individually or together, are not known to adversely affect kidney function [18–20], and serum calcium levels were within the normal range. It is likely that microcalcifications were the result of dystrophic calcification of cells damaged by a previously unknown insult and not an indication of a primary role for calcium [11].

Most of the patients with ATN in our current study had histologic evidence of chronic injury. Patients with community acquired AKI have up to three times the rate of ESRD as the general population, with the ESRD being primarily related to advanced age and high rates of cardiovascular disease [21, 22]. Whether the risk applies to younger patients is not clear, but baseline normal renal function associates with a decreased risk of ESRD over time [22, 23]. This implies that the prognosis in otherwise healthy young men will not be compromised if the injury is not repeated.

In some cases, the pathology in bodybuilders has been a TIN resembling a drug-type allergy in which the renal failure resolves when supplements are discontinued [24, 25]. This TIN is uncommon and, in the current study, occurred at an annual rate of 1 per 200,000 gymnasium users, a frequency not different than TIN in the general population.

For the level of anabolic steroid use practiced in the region that includes multiple drug regimens in 21% of experienced bodybuilders, the incidence of FSGS could not be considered any greater than that of the general population. While there is experimental evidence that anabolic steroids may be toxic to podocytes [7–9], the primary association between anabolic steroids and FSGS comes from case studies and particularly the 2011 report by Herlitz et al. [8] of 10 bodybuilders aged 28 to 45 years old that developed FSGS after years of training that included using multiple anabolic steroids. This cohort consisted of nine patients from New York City (NYC) and one from Boston that was collected over a 10 year period at three major reference centers and presumably reflects the collective experience of nephrologists and pathologists in the region. A similar 2018 paper by El-Reshaid et al. [26], reported FSGS among eight “elite” anabolic steroid using bodybuilders from Kuwait that were collected over a period of 5 years.

The use of anabolic steroids among males in NYC is probably similar to the 2–4% that is estimated for the US as a whole [27, 28]. In this case, the population of anabolic steroid users in the 2011 NYC population of 1.65 million males 20–49 years old would be approximately 31,400 [29]. The nine NYC patients in the paper by Herlitz et al. [8] would then calculate to an annual ASIR of 2.9 (1.0 to 4.8) patients per 100,000 steroid users.

The incidence of FSGS has been estimated from biopsy series from Olmstead County, Minnesota and from Melbourne, Australia [30, 31]. The FSGS estimate of 2.9 (1.0 to 4.8) per 100,000 NYC body builders may indicate an increased risk when compared to the all age and both gender FSGS incidence of 1.1 (0.7 to 1.5) per 100,000 persons in Olmstead County [30]. It does not indicate a significantly increased risk when compared to the FSGS rate of 1.9 (1.3 to 2.5) per 100,000 white males 25–44 years old in Melbourne [31]. The population of Kuwait is similar to Iraqi Kurdistan, and the biopsy frequency of 1.6 cases of FSGS per year among anabolic steroid using bodybuilders reported by El-Reshaid et al. [26] would certainly seem excessive, but a relationship to the general incidence of FSGS and the chance of episodic random clustering among bodybuilders must also be considered.

One difficulty of making a comparison between most bodybuilders and the patients reported by Herlitz et al. [8] and El-Reshaid et al. [26] is that the NYC and Kuwaiti bodybuilders would be considered steroid dependent, a condition estimated to afflict about 30% of anabolic steroid users but probably not a factor in Kurdistan [27, 28]. It is not at all clear whether the FSGS anabolic steroid risk should be based upon all users or only those considered dependent. Although the number of patients may be too small for the detection of rare kidney events, clinical studies of dependent anabolic steroids users have found “accelerated” coronary atherosclerosis and left ventricular muscle dysfunction but have not mentioned renal disease [27, 28].

Nevertheless, if FSGS is increased among US anabolic steroid users, dependent or otherwise, the frequency of its recognition seems disproportionately low compared to the high rates of anabolic steroid exposure in US athletic communities [5, 27, 28, 32, 33]. In a 2019 scientific statement, the Endocrine Society recognized FSGS as a complication of anabolic steroid use, but considered it uncommon and less serious than cardiovascular disease [28].

Nearly all of the reports of nephrocalcinosis complicating bodybuilding have come from Brazil [2–4], but the use of up to 10,000 units a day of vitamin D is recommended in US and European muscle building e-magazines as a “steroid” that enhances muscle development [34, 35]. With this level of advocacy, it is difficult to understand why nephrocalcinosis among bodybuilders appears to be so regionally localized, but it may be the method of delivery that contributes to the disease.

The oil-based veterinary compounds are inexpensive and mainly used to add bulk to specific muscle groups. As was found in patient 15, the granulomatous oil containing reaction can act as a slowly releasing reservoir for the lipid soluble vitamins for months and possibly years [36]. While the injection of high-dose veterinary vitamin compounds does not seem to have any role in Western bodybuilding, some European bodybuilders inject paraffin oils around muscles for their contouring effect, a practice that is also seen in some cosmetic surgeries [37, 38]. The oils elicit a granulomatous reaction that is associated with hypercalcemia as a result of the local synthesis of active vitamin D [36, 37]. Renal failure that is corrected when calcium and vitamin D levels are lowered is reported in many of these patients [4, 36, 37].

The limitations of our current study include the accuracy of estimating gymnasium participation and supplement use. Such estimates necessarily require sampling and self reporting since it is not possible to directly censor these factors. Our bodybuilding estimates were derived from data provided by large regional gymnasiums, and queries about supplement use were made only in Sulaimania. Data could be biased towards large gymnasiums and single city supplement use, but reviewing officials stated that the findings reflected their experience and that little variation would be expected in the relatively homogeneous Kurdistan region.

The principal limitation was that kidney disease was uncommon among gymnasium participants. This is inherent in the evaluation of any type of rare disease where events may cluster or go undetected for extended periods of time [13]. FSGS is a useful example because of its controversial association with anabolic steroids. We estimate that a relationship between FSGS and anabolic steroids in our region would have required the identification of seven FSGS patients over the 9.0 year collection period to be considered significantly different than its usual population frequency (Table 4). The threshold of seven patients is needed despite what appears to be marked increases in ASIR with a simulated increase of even three or four FSGS patients.

**Table 4:** Projected age specific incidence rates (ASIR) for focal segmental glomerulosclerosis with increasing numbers of biopsy diagnoses

Diagnosis	Biopsies 9 yrs	Annual average	Pop at risk	ASIR	95% CI
FSGS steroid users	2	0.22	6500	3.4	−1.3 to 8.1
“	3	0.33	6500	5.1	−0.7 to 10.8
“	4	0.44	6500	6.8	0.1 to 13.4
“	5	0.56	6500	8.6	1.1 to 16.2
“	6	0.67	6500	10.3	2.1 to 18.6
“	7	0.78	6500	12.0	3.1 to 20.9
FSGS gen population	–	22.5	1,100,000	2.0	1.2 to 2.8

Rates are for anabolic steroid using bodybuilders are compared with FSGS rates in the male general population of Kurdistan of Iraq aged 15 to 39 years for the years 2012–2013. ASIR is calculated per 100,000 males

The calculations emphasize that comparisons of the frequency of rare events that are typical of most kidney diseases can be misleading and require a measure of statistical uncertainty. In population studies, this is usually achieved by confidence intervals, but because rare events produce very wide confidence intervals, the relevance of the estimates can be difficult to understand [13].

It is also a concern that our interest in bodybuilding-related kidney disease may have created an investigative bias, as the biopsy frequency for regular gymnasium participants was more than twice that of the general population. Since, however, the different biopsy frequencies uncovered essentially the same rates of disease, it is likely that, except for nephrocalcinosis, the kidney health of bodybuilders is not worse than that of other young men in the region.

## Conclusion

Young middle-Eastern men participate in bodybuilding and consume supplements including anabolic steroids like their counterparts in the US and Europe. In this population-based biopsy study, we found that the frequency of kidney disease among Kurdistan Iraqi bodybuilders as measured by age-specific incidence rates was not significantly different than the age matched general population with one important exception. Persons who injected veterinary grade vitamin D compounds for their muscle contouring effect assumed a high risk of end-stage kidney disease. These injections have been recently introduced into the region and enjoy some popularity. The practice is not condoned by gymnasium trainers or managers and needs to be addressed by health authorities as having substantial morbidity and potential mortality.

## Supplementary information

**Additional file 1.**

## Data Availability

Compiled data and calculations are stored in Excel files in the Shorsh University Hospital Pathology Department and will be made available upon request to the corresponding author MDH.

## Abbreviations

FSGS: Focal segmental glomerulosclerosis
MGN: Membranous glomerulonephritis
TIN: Tubulointerstitial nephritis
PSGN: Post-streptococcal glomerulonephritis
ATN: Acute tubular necrosis
AKI: Acute kidney injury
ASIR: Age standardized incidence rate
95% CI: 95% confidence intervals
ESRD: End-stage renal disease
IF/TA: Interstitial fibrosis and tubular atrophy
NYC: New York City
