# Supplementary material for: Effects of bodybuilding supplements on the kidney: A population-based incidence study of biopsy pathology and clinical characteristics among middle eastern men
Source: BMC Nephrol. 2020 May 6;21:164. doi: 10.1186/s12882-020-01834-5 (PMC7203829; doi:10.1186/s12882-020-01834-5)
Supplement: Supplementary file 1 — Additional file 1. [file 12882_2020_1834_MOESM1_ESM.docx]

Questionaire for body building supplements

Gym name:_______________

Code number of athlete:_______________

Age:________________

Length of time participating in body building:_____________

Do you use any of the following supplements:

Protein powder:____________, amount per day:_____________

Creatine powder:____________, amount per day:_______________

Vitamins:______, if oral , number of tablets/capsules per day________

If injected vitamins, number of injections per month:________

Have you ever injected veterinary Vit A D3 E (ADE, or AD3):______, How many times:________

Anabolic steroids: circle product, add another if not listed:____________

Dianabol, Dbol, Boldinon, alphabol, terenbolon, times per month:________

Nandrolol (deca durabolin), times per month:_________

Dynobolon, Psychobolan, times per month:__________

Sustanon, times per month:_________

Testosterone:_______, times per month:________

Winstrol, times per month

Do you do steroid cycling:________(list products used):_____________________

Do you inject growth hormone (HGH):________, times per month________

Do you use any of the following

Animal M-Stak:__________

Animal PAK:___________

Optimen:__________

Body Lab:_________

Mass Gainer:________

Do you use energy drinks: While training:_________, number per day:________

I Acknowledge that I am participating in a study of the effects of body building supplements on my kidneys, and that the information may be used for publication in medical journals and for making decisions on public health policies.

Please indicate that you have read and understand this acknowledgement with your code number: _________
